# Au(I)-Catalyzed Annulation of Propargyl Amine with Aldehydes: One-Pot Cascade Synthesis of 2,5-Dimethylpyrazines

**DOI:** 10.3390/ijms16023599

**Published:** 2015-02-05

**Authors:** Ji Su, Huixin Liu, Ruimao Hua

**Affiliations:** 1 Department of Chemistry, Tsinghua University, Beijing 100084, China; E-Mails: andrew-su@163.com (J.S.); liuhuixin9015@163.com (H.L.); 2 National Engineering Laboratory for Rice and Byproduct Deep Processing, Central South University of Forestry and Technology, Changsha 410004, China

**Keywords:** aldehydes, annulation, golden complex, propargyl amine, pyrazines

## Abstract

3-Substituted 2,5-dimethylpyrazines were synthesized in high yields via a one-pot cascade annulation of easily available propargyl amine with aldehydes catalyzed by Au(PPh_2_Cy)Cl.

## 1. Introduction

**Scheme 1:**
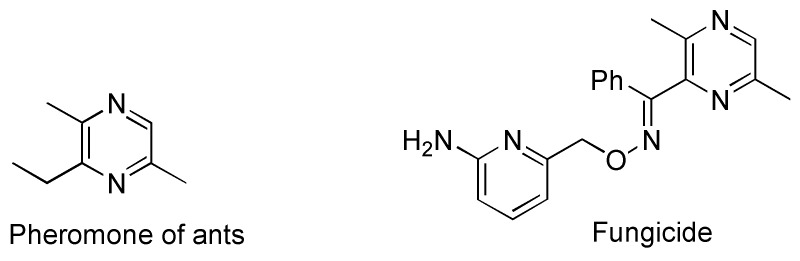
Examples of natural products having structural unit of 2,5-dimethylpyrazine (DMP).

Transition-metal-catalyzed cyclization of alkynes with nitrogen-containing compounds has provided the efficient synthetic methods for *N*-heterocyclic compounds [1,2,3,4]. With the interest of developing the efficient procedures approach to *N*-heterocyclic compounds, we have recently studied the cyclization of alkynes or 1,3-butadiynes with various nitrogen-containing compounds affording 1,2,5-trisubsituted pyrroles [5], isoquinolines [6,7], 2,4,6-triarylpyridines [8], benzo[*f*]quinazolines [9], indoles [10], ring-fused phenanthroimidazoles [11], and 1,2,4-oxadiazoles [12]. On the other hand, cyclic compounds containing the structural unit of 2,5-dimethylpyrazine (DMP) show interesting physiological and biological activities found to be the pheromone of ants [13,14], and fungicide active agents [15] (Scheme 1). In addition, the structural unit of DMP has become increasingly important applications as versatile ligands in the field of supramolecular chemistry due to their coordinative ability of two symmetric nitrogen atoms [16,17,18,19,20].

**Scheme 2:**
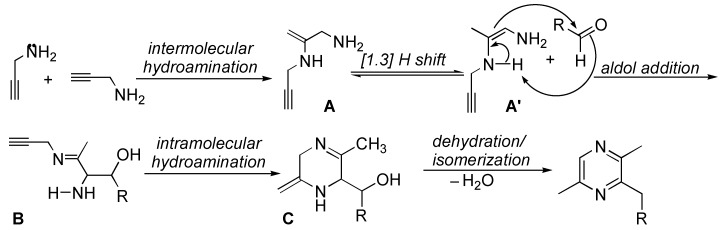
Proposed mechanism for the formation of pyrazine’s ring.

It has been well documented that propargylic compounds, such as propargyl amines [21,22], and propargyl alcohols [23,24,25] have been widely applied as one of the important building blocks in the synthesis of a variety of heterocyclic compounds containing the relevant heteroatoms. As a continuation of our interest in the applications of propargylic compounds on the synthesis of heterocyclic compounds [23,24,25], we are interested in exploring the possible application of prop-2-yn-1-amine (a simplest molecule of propargyl amine) in the synthesis of DMP. Therefore, we designed a synthetic protocol for the formation of DMP as shown in Scheme 2. It involves the dimerization of propargyl amines via the hydroamination to give α-amino enamine **A** and its rearranged isomer **A'**, which serves as a nucleophile to undergo the aldol addition with aldehyde to form α-amino imine intermediate **B** [26]. The subsequent intramolecular hydroamination and dehydration/isomerization form cyclic structure of pyrazine. After we developed the catalytic system and finished the experiments [27], a similar procedure for the formation of pyrazine ring was recently reported [28].

## 2. Results and Discussion

We initiated our investigation on the reaction of prop-2-yn-1-amine with benzaldehyde (**1a**) in presence of Au(I) complexes, since Au(I) complexes have been found to be the efficient catalysts for the intermolecular [29,30] and intramolecular hydroamination of alkynes [31,32,33], as well as cycloisomerization of alkynes [34,35] to give *N*-heterocyclic compounds. As concluded in Table 1, when a mixture of benzaldehyde (1.0 mmol, **1a**) and propargyl amine (3.0 equiv) and Au(PPh_3_)Cl (0.05 mmol) in toluene was heated with stirring at 60 °C for 48 h, the analyses of the reaction mixture by GC-MS revealed that a new dehydrative cyclization of one molecule of **1a** with two molecules of propargyl amine occurred to produce 3-benzyl-2,5-dimethylpyrazine (**2a**) in 13% GC yield (entry 1). The formation of **2a** greatly depended on the solvents used. For example, when THF was used to lead to no formation of **2a** at all (entry 2). However, the yield of **2a** could be substantially increased to 45%, when CH_3_CN was employed (entry 3). Increasing the reaction temperature to 80 °C in CH_3_CN resulted in the reaction much more efficiently to afford **2a** in 77% yield (entry 4), and the almost quantitative yields of **2a** could be obtained by simply replacing PPh_3_ ligand to PPh_2_Me (entry 5) or PPh_2_Cy (entry 6) in CH_3_CN at 60 °C. In the presence of Au(PPh_2_Cy)Cl, repeating the reaction in toluene (entry 7) and THF (entry 8) resulted in low yield or no formation of **2a**. In addition, Au(PPhMe_2_)Cl and Au(PCy_3_)Cl also showed good catalytic activities to give **2a** in 72% (entry 9) and 88% GC (entry 10) yields, respectively. It should be noted that reduction of reaction time to 24 h led to the slight decrease of the yields of **2a** in the cases of Au(PPh_2_Me)Cl (entry 11, 89%) and Au(PPh_2_Cy)Cl (entry 12, 92%) used.

**Table 1:** Optimizing the reaction conditions for the formation of 3-benzyl-2,5-dimethylpyrazine (**2a**) *^a^*.

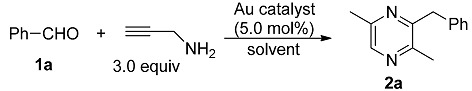				
Entry	Catalyst	Solvent	Temp. (°C)/Time (h)	Yield (%) *^b^*
1	Au(PPh_3_)Cl	toluene	60/48	13
2	Au(PPh_3_)Cl	THF	60/48	0
3	Au(PPh_3_)Cl	CH_3_CN	60/48	45
4	Au(PPh_3_)Cl	CH_3_CN	80/48	77
5	Au(PPh_2_Me)Cl	CH_3_CN	60/48	>99
6	Au(PPh_2_Cy)Cl	CH_3_CN	60/48	>99 (92)
7	Au(PPh_2_Cy)Cl	toluene	60/48	20
8	Au(PPh_2_Cy)Cl	THF	60/48	0
9	Au(PPhMe_2_)Cl	CH_3_CN	60/48	72
10	Au(PCy_3_)Cl	CH_3_CN	60/48	88
11	Au(PPh_2_Me)Cl	CH_3_CN	60/24	89
12	Au(PPh_2_Cy)Cl	CH_3_CN	60/24	92

*^a^* Reactions were carried out using 1.0 mmol of benzaldehyde (**1a**), 3.0 mmol of pro-2-yn-1-amine, and 0.05 mmol of catalyst in 2.0 mL of solvent in a sealed tube under nitrogen atmosphere; *^b^* GC yield based on the amount of **1a** used. Number in parenthesis is isolated yield.

With the optimized reaction condition indicated in entry 6 of Table 1, the generality for the formation of 3-substituted 2,5-dimethylpyrazines was studied. As shown in Table 2, benzaldehydes bearing chloro group at *para*-, *meta*- or *ortho*-position, or having bromo, fluoro, methyl or methoxy group at *para*-position reacted with propargyl amine smoothly to afford the corresponding pyrazines **2b**–**f** and **2h**–**i** in high yields. No significant steric effect was observed when *para*-chlorobenzaldehyde (for **2b**), *meta*-chlorobenzaldehyde (for **2c**) and *ortho*-chlorobenzaldehyde (for **2d**) were used, and the desired products **2b**–**d** were obtained in similar yields. By comparison of the reactions in the cases of *para*-chlorobenzaldehyde (for **2b**), *para*-fluorobenzaldehyde (for **2e**), *para*-methylbenzaldehyde (for **2h**) and *para*-methoxybenzaldehyde (for **2i**) used, the electron effect of substitute groups could not affect the formation of the corresponding pyrazines in high yields either. Only in the case of 2,4-dichlorobenzaldehyde employed, the corresponding product was formed in a declined yield (**2g**, 73%). In addition, it was very important to note that under the reaction conditions, C–X bond (X = F, Cl, Br) remained intact, and the obtained 3-arylmethyl-2,5-dimethylpyrazines can be easily transferred into their new derivatives by C–X bond activation and its coupling reaction. In addition, we also examined the present cyclization employing aliphatic aldehydes, and the reactions occurred smoothly to afford the corresponding desired pyrazines (**2k**–**m**) with high yields.

**Table 2:** Synthesis of 2,5-dimethylpyrazine derivatives ^a^.

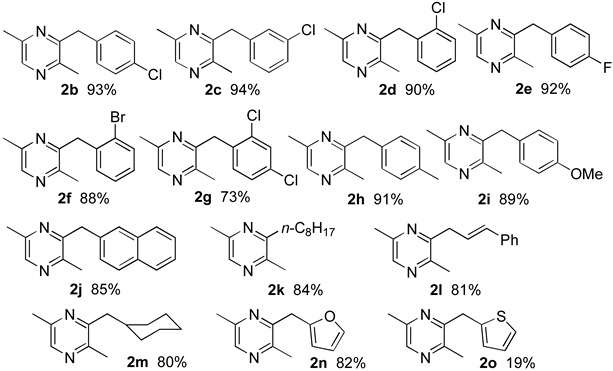

^a^ Reactions were carried out using 2.0 mmol of aldehyde, 6.0 mmol of prop-2-yn-1-amine, and 0.1 mmol of catalyst in 4.0 mL of MeCN at 60 °C for 48 h.

Moreover, the annulation of propargyl amine with heterocyclic aldehydes such as 2-furaldehyde and 2-thiophenaldehyde were also studied, and in the case of 2-furaldehyde used, the corresponding 2,5-dimethylpyrazine (**2n**) was obtained in high yield. However, when 2-thiophenaldehyde was subjected to the similar reaction conditions, the desired product (**2o**) formed in 19% isolated yield, accompanied with the formation of *N*-(prop-2-yn-1-yl)-1-thiophen-2-ylmethanimine in 70% yield resulting from the traditional nucleophilic addition of propargyl amine to aldehyde and subsequent dehydration reaction.

However, unfortunately, the reactions of **1a** or **1k** with 3-substituted propargyl amines such as 3-phenyl-2-propyn-1-amine and 2-heptyn-1-amine resulted in neither affording the corresponding pyrazine derivatives, nor forming other *N*-heterocyclic compounds.

## 3. Experimental Section

### 3.1. General Methods

All organic starting materials and solvents are analytically pure and used without further purification. Nuclear magnetic resonance (NMR) spectra were recorded on a JEOL ECA-300 spectrometer (JEOL, Tokyo, Japan) using CDCl_3_ as a solvent at 298 K. ^1^H NMR (300 MHz) chemical shifts (δ) were referenced to internal standard TMS (for ^1^H, δ = 0.00 ppm). ^13^C NMR (75 MHz) chemical shifts were referenced to internal solvent CDCl_3_ (for ^13^C, δ = 77.16 ppm). Mass spectra (MS) were obtained on a Shimadzu GCMS-QP2010S (Shimadzu, Tokyo, Japan), and high-resolution mass spectra (ESI) were obtained with a micrOTOF-Q 10142 spectrometer (Agilent, San Diego, CA, USA).

### 3.2. A Typical Experiment Procedure for the Reaction of Benzaldehyde (**1a**) with Prop-2-yn-1-amine Affording 3-Benzyl-2,5-dimethylpyrazine (**2a**) (Table 1, Entry 6)

A mixture of benzaldehyde (**1a**) (106.0 mg, 1.0 mmol), prop-2-yn-1-amine (165.0 mg, 3.0 mmol), Au(PPh_2_Cy)Cl (25.0 mg, 0.05 mmol) and CH_3_CN (2.0 mL) was heated at 60 °C (oil bath temperature) with stirring for 48 h in a screw-capped thick-walled Pyrex tube under a nitrogen atmosphere. After the reaction mixture was cooled to room temperature, CH_2_Cl_2_ (3.0 mL) and *n*-octadecane (51.0 mg, 0.2 mmol as internal standard for GC analysis) was then added with stirring. After GC and GC-MS analyses of the reaction mixture, volatiles were removed under reduced pressure, and the residue was subjected to silica gel column chromatography (silica gel was alkalized by a solution of petroleum ether with 2% (*v*/*v*) triethylamine), eluted with a mixture of solvents of triethylamine/ethyl acetate/petroleum ether (1:20:80 in volume). **2a** was obtained in 182.0 mg (0.92 mmol, 92%) as a yellow oil. The GC analysis of reaction mixture disclosed the formation of **2a** in >99% GC yield.

Characterization data of products (the charts of ^1^H- and ^13^C-NMR are reported in Supplementary Materials):

**3-Benzyl-2,5-dimethylpyrazine (2a) [36]:** yellow oil; ^1^H NMR (300 MHz, CDCl_3_) δ 8.20 (s, 1H), 7.28–7.15 (m, 5H), 4.15 (s, 2H), 2.51 (s, 3H), 2.43 (s, 3H); ^13^C NMR (75 MHz, CDCl_3_) δ 152.9, 150.1, 149.3, 141.4, 137.9, 128.6, 128.4, 126.4, 41.6, 21.4, 21.0; GCMS *m*/*z* (% rel. intensity) 198 (M^+^, 66), 197 (100), 183 (40), 128 (8), 91 (14); HRMS (ESI): Calcd. for C_13_H_15_N_2_ [M + H]^+^: 199.1230; found: 199.1232.

**3-(4-Chlorobenzyl)-2,5-dimethylpyrazine (2b):** yellow oil; ^1^H NMR (300 MHz, CDCl_3_) δ 8.22 (s, 1H), 7.22 (d, 2H, *J* = 8.6 Hz), 7.11 (d, 2H, *J* = 8.3 Hz), 4.11 (s, 2H), 2.51 (s, 3H), 2.43 (s, 3H); ^13^C NMR (75 MHz, CDCl_3_) δ 153.3, 150.3, 149.1, 141.5, 136.4, 132.2, 129.9, 128.5, 40.8, 21.3, 21.0; GCMS *m*/*z* (% rel. intensity) 233 (44), 232 (M^+^, 79), 231 (100), 217 (34), 197 (23), 196 (24), 182 (17); HRMS (ESI): Calcd. for C_13_H_14_ClN_2_ [M + H]^+^: 233.0840; found: 233.0844.

**3-(3-Chlorobenzyl)-2,5-dimethylpyrazine (2c):** yellow oil; ^1^H NMR (300 MHz, CDCl_3_) δ 8.23 (s, 1H), 7.25–7.12 (m, 3H), 7.07 (m, 1H), 4.13 (s, 2H), 2.52 (s, 3H), 2.45 (s, 3H); ^13^C NMR (75 MHz, CDCl_3_) δ 152.1, 150.4, 149.2, 141.7, 140.0, 134.3, 129.7, 128.6, 126.8, 126.6, 41.1, 21.4, 21.1; GCMS *m*/*z* (% rel. intensity) 233 (41), 232 (M^+^, 76), 231 (100), 217 (47), 197 (24), 196 (27), 182 (19), 116 (22); HRMS (ESI): Calcd. for C_13_H_14_ClN_2_ [M + H]^+^: 233.0840; found: 233.0847.

**3-(2-Chlorobenzyl)-2,5-dimethylpyrazine (2d):** yellow oil; ^1^H NMR (300 MHz, CDCl_3_) δ 8.24 (s, 1H), 7.38 (d, 1H, *J* = 7.9 Hz), 7.20–7.08 (m, 2H), 6.88 (d, 1H, *J* = 7.2 Hz), 4.27 (s, 2H), 2.50 (s, 3H), 2.43 (s, 3H); ^13^C NMR (75 MHz, CDCl_3_) δ 152.0, 150.4, 149.6, 141.6, 136.0, 134.1, 129.9, 129.4, 127.8, 126.8, 38.6, 21.3, 21.1; GCMS *m*/*z* (% rel. intensity) 197 (100), 116 (8), 89 (7); HRMS (ESI): Calcd. for C_13_H_14_ClN_2_ [M + H]^+^: 233.0840; found: 233.0838.

**3-(4-Fluorobenzyl)-2,5-dimethylpyrazine (2e):** yellow oil; ^1^H NMR (300 MHz, CDCl_3_) δ 8.22 (s, 1H), 7.20–2.09 (m, 2H), 6.97–6.90 (m, 2H), 4.12 (s, 2H), 2.52 (s, 3H), 2.44 (s, 3H); ^13^C NMR (75 MHz, CDCl_3_) δ 161.5 (d, *J*_C–F_ = 242.4 Hz), 152.7, 150.3, 149.1, 141.5, 133.6 (d, *J*_C–F_ = 2.9 Hz), 130.0 (d, *J*_C–F_ = 7.9 Hz), 115.2 (d, *J*_C–F_ = 20.8 Hz), 40.7, 21.3, 21.0; GCMS *m/z* (% rel. intensity) 216 (M^+^, 71), 215 (100), 201 (38), 109 (22); HRMS (ESI): Calcd. for C_13_H_14_FN_2_ [M + H]^+^: 217.1136; found: 217.1126.

**3-(2-Bromobenzyl)-2,5-dimethylpyrazine (2f):** yellow solid; ^1^H NMR (300 MHz, CDCl_3_) δ 8.24 (s, 1H), 7.56 (d, 1H, *J* = 6.5 Hz), 7.20–7.01 (m, 2H), 6.84 (d, 1H, *J* = 7.6 Hz), 4.26 (s, 2H), 2.49 (s, 3H), 2.43 (s, 3H); ^13^C NMR (75 MHz, CDCl_3_) δ 151.9, 150.3, 149.6, 141.6, 137.7, 132.7, 129.9, 128.0, 127.4, 124.7, 41.3, 21.3, 31.1; GCMS *m*/*z* (% rel. intensity) 197 (M–Br^−^, 100), 154 (5), 128 (10), 89 (9), 63 (5); HRMS (ESI): Calcd. for C_13_H_14_BrN_2_ [M + H]^+^: 277.0335; found: 277.0327.

**3-(2,4-Dichlorobenzyl)-2,5-dimethylpyrazine (2g):** yellow oil; ^1^H NMR (300 MHz, CDCl_3_) δ 8.24 (s, 1H), 7.40 (s, 1H), 7.12 (d, 1H, *J* = 7.5 Hz), 6.86 (d, 1H, *J* = 8.2 Hz), 4.21 (s, 2H), 2.49 (s, 3H), 2.44 (s, 3H); ^13^C NMR (75 MHz, CDCl_3_) δ 151.5, 150.6, 149.5, 141.8, 134.7, 133.5, 132.9, 131.0, 129.2, 127.1, 38.0, 21.3, 21.1; GCMS *m*/*z* (% rel. intensity) 231 (M–Cl^−^, 100), 196 (84), 150 (8), 80 (8), 51 (5); HRMS (ESI): Calcd. for C_13_H_13_Cl_2_N_2_ [M + H]^+^: 267.0450; found: 267.0457.

**3-(4-Methylbenzyl)-2,5-dimethylpyrazine (2h):** yellow oil; ^1^H NMR (300 MHz, CDCl_3_) δ 8.18 (s, 1H), 7.10–7.00 (m, 4H), 4.10 (s, 2H), 2.49 (s, 3H), 2.42 (s, 3H), 2.25 (s, 3H); ^13^C NMR (75 MHz, CDCl_3_) δ 152.9, 149.8, 149.0, 141.0, 135.6, 134.6, 128.9, 128.3, 41.0, 21.2, 20.8, 20.7; GCMS *m*/*z* (% rel. intensity) 212 (M^+^, 75), 211 (100), 197 (53), 128 (11), 105 (30), 77 (16); HRMS (ESI): Calcd. for C_14_H_17_N_2_ [M + H]^+^:213.1385; found: 213.1385.

**3-(4-Methoxybenzyl)-2,5-dimethylpyrazine (2i):** yellow solid; ^1^H NMR (300 MHz, CDCl_3_) δ 8.20 (s, 1H), 7.10 (d, 2H, *J* = 8.3 Hz), 6.81 (d, 2H, *J* = 8.2 Hz), 4.10 (s, 2H), 3.75 (s, 3H), 2.52 (s, 3H), 2.44 (s, 3H); ^13^C NMR (75 MHz, CDCl_3_) δ 158.2, 153.3, 150.2, 149.3, 141.3, 130.0, 129.6, 113.9, 55.2, 40.9, 21.5, 21.1; GCMS *m*/*z* (% rel. intensity) 228 (M^+^, 100), 227 (44), 213 (74), 185 (17), 121 (87), 91 (14); HRMS (ESI): Calcd. for C_14_H_17_N_2_O [M + H]^+^: 229.1335; found: 229.1330.

**3-(2-Naphthylmethyl)-2,5-dimethylpyrazine (2j):** yellow oil; ^1^H NMR (300 MHz, CDCl_3_) δ 8.19 (s, 1H), 7.75–7.66 (m, 3H), 7.53 (s, 1H), 7.41–7.30 (m, 3H), 4.27 (s, 2H), 2.50 (s, 3H), 2.43 (s, 3H); ^13^C NMR (75 MHz, CDCl_3_) δ 152.7, 150.1, 149.4, 141.4, 135.4, 133.4, 132.1, 128.1, 127.5, 127.4, 127.0, 126.8, 126.0, 125.4, 41.7, 21.4, 21.0; GCMS *m*/*z* (% rel. intensity) 248 (M^+^, 85), 247 (100), 233 (42), 141 (29), 115 (27); HRMS (ESI): Calcd. for C_17_H_17_N_2_ [M + H]^+^: 249.1386; found: 249.1392.

**3-Octyl-2,5-dimethylpyrazine (2k):** yellow oil; ^1^H NMR (300 MHz, CDCl_3_) δ 8.15 (s, 1H), 2.76 (t, 2H, *J* = 7.6 Hz), 2.53 (s, 3H), 2.49 (s, 3H), 1.73–1.62 (m, 2H), 1.48–1.21 (m, 10H), 0.88 (t, 3H, *J* = 6.5 Hz); ^13^C NMR (75 MHz, CDCl_3_) δ 154.9, 150.0, 148.5, 140.6, 35.2, 31.9, 29.7, 29.5, 29.3, 28.6, 22.7, 21.2, 21.1, 14.1; GCMS *m*/*z* (% rel. intensity) 135 (11), 122 (100); HRMS (ESI): Calcd. for C_14_H_25_N_2_ [M + H]^+^: 221.2012; found: 221.2019.

**3-Cinnamyl-2,5-dimethylpyrazine (2l):** orange oil; ^1^H NMR (300 MHz, CDCl_3_) δ 8.20 (s, 1H), 7.35–7.20 (m, 5H), 6.50–6.31 (m, 2H), 3.71 (d, 2H, *J* = 5.8 Hz), 2.55 (s, 3H), 2.50 (s, 3H); ^13^C NMR (75 MHz, CDCl_3_) δ 152.4, 150.4, 149.1, 141.4, 137.2, 131.9, 128.5, 127.4, 126.2, 125.9, 39.2, 21.2, 21.1; GCMS *m*/*z* (% rel. intensity) 224 (M^+^, 47), 223 (28), 209 (34). 147 (45), 122 (100), 115 (27), 91 (15); HRMS (ESI): Calcd. for C_15_H_17_N_2_ [M + H]^+^: 225.1386; found: 225.1375.

**3-Cyclohexylmethyl-2,5-dimethylpyrazine (2m):** yellow oil; ^1^H NMR (300 MHz, CDCl_3_) δ 8.14 (s, 1H), 2.66 (d, 2H, *J* = 7.2 Hz), 2.52 (s, 3H), 2.50 (s, 3H), 1.85–1.63 (m, 6H), 1.29–0.99 (m, 5H); ^13^C NMR (75 MHz, CDCl_3_) δ 154.1, 150.0, 149.0, 140.6, 42.5, 38.3, 33.3, 26.5, 26.3, 21.6, 21.2; GCMS *m*/*z* (% rel. intensity) 204 (M^+^, 0.2), 189 (1), 161 (2), 147 (2), 122 (100), 80 (2), 55 (4); HRMS (ESI): Calcd. for C_13_H_21_N_2_ [M + H]^+^: 205.1699; found: 205.1702.

**3-(2-Furylmethyl)-2,5-dimethylpyrazine (2n):** yellow oil; ^1^H NMR (300 MHz, CDCl_3_) δ 8.23 (s, 1H), 7.31 (d, 1H, *J* = 1.1 Hz), 6.28 (d, 1H, *J* = 1.5 Hz), 6.00 (d, 1H, *J* = 3.1 Hz), 4.16 (s, 2H), 2.53 (s, 3H), 2.51 (s, 3H); ^13^C NMR (75 MHz, CDCl_3_) δ 151.7, 150.6, 150.4, 149.4, 141.9, 141.7, 110.4, 106.7, 35.0, 21.3, 21.1; GCMS *m*/*z* (% rel. intensity) 188 (M^+^, 80), 159 (100), 145 (15), 91 (10), 81 (36); HRMS (ESI): Calcd. for C_11_H_17_N_2_O [M + H]^+^: 189.1022; found: 189.1017.

**2,5-Dimethyl-3-(2-thienylmethyl)pyrazine (2o):** yellow oil; ^1^H NMR (300 MHz, CDCl_3_) δ 8.22 (s, 1H), 7,13 (d, 1H, *J* = 6.5 Hz), 6.90 (dd, 1H, *J* = 3.4 Hz, 5.1 Hz), 6.78 (m, 1H), 4.32 (s, 2H), 2.52 (s, 6H); ^13^C NMR (75 MHz, CDCl_3_) δ 152.1, 150.4, 149.0, 141.8, 140.4, 126.8, 125.4, 124.3, 36.2, 21.3, 21.1; GCMS *m*/*z* (% rel. intensity) 204 (100), 189 (15), 171 (30), 159 (35), 97 (95), 80 (9), 53 (16); HRMS (ESI): Calcd. for C_11_H_13_N_2_S [M + H]^+^: 205.0794; found: 205.0790.

## 4. Conclusions

In summary, we have developed a cascade annulation of propargyl amine with aldehydes approach to 3-substituted 2,5-dimethylpyrazines in high yields catalyzed by Au(PPh_2_Cy)Cl, which involves the intermolecular hydroamination and intramolecular cyclic hydroamination, as well as the dehydration reaction. The present work has developed the application of propargyl amines in the synthesis of nitrogen-containing heterocycles with the advantages of readily accessible starting materials and high atom-efficiency.

## Supplementary Materials

Supplementary materials can be found at http://www.mdpi.com/1422-0067/16/02/3599/s1.
